# Does a reduced glucose intake prevent hyperglycemia in children early after cardiac surgery? a randomized controlled crossover study

**DOI:** 10.1186/cc11658

**Published:** 2012-10-02

**Authors:** Carlijn TI de Betue, Sascha CAT Verbruggen, Henk Schierbeek, Shaji K Chacko, Ad JJC Bogers, Johannes B van Goudoever, Koen FM Joosten

**Affiliations:** 1 Intensive Care and Department of Pediatric Surgery, Erasmus MC - Sophia Children's Hospital, University Medical Center Rotterdam, Dr. Molewaterplein 60, 3015 GJ, Rotterdam, The Netherlands; 2 Department of Pediatrics, Erasmus MC - Sophia Children's Hospital, University Medical Center Rotterdam, Dr. Molewaterplein 60, 3015 GJ, Rotterdam, The Netherlands; 3 Department of Pediatrics, Emma Children's Hospital, Academic Medical Center, University of Amsterdam, Meibergdreef 9. 1105 AZ, Amsterdam, The Netherlands; 4 Department of Pediatrics, Baylor College of Medicine, USDA-ARS Children's Nutrition Research Center, 1100 Bates Street, Houston, TX 77030, USA; 5 Department of Cardiothoracic Surgery, Erasmus MC, University Medical Center Rotterdam, Dr. Molewaterplein 50, 3015 GE, Rotterdam, The Netherlands; 6 Department of Pediatrics, VU University Medical Center, De Boelelaan 1117, 1081 HV, Amsterdam, The Netherlands

## Abstract

**Introduction:**

Hyperglycemia in children after cardiac surgery can be treated with intensive insulin therapy, but hypoglycemia is a potential serious side effect. The aim of this study was to investigate the effects of reducing glucose intake below standard intakes to prevent hyperglycemia, on blood glucose concentrations, glucose kinetics and protein catabolism in children after cardiac surgery with cardiopulmonary bypass (CPB).

**Methods:**

Subjects received a 4-hour low glucose (LG; 2.5 mg/kg per minute) and a 4-hour standard glucose (SG; 5.0 mg/kg per minute) infusion in a randomized blinded crossover setting. Simultaneously, an 8-hour stable isotope tracer protocol was conducted to determine glucose and leucine kinetics. Data are presented as mean ± SD or median (IQR); comparison was made by paired samples *t *test.

**Results:**

Eleven subjects (age 5.1 (20.2) months) were studied 9.5 ± 1.9 hours post-cardiac surgery. Blood glucose concentrations were lower during LG than SG (LG 7.3 ± 0.7 vs. SG 9.3 ± 1.8 mmol/L; *P *< 0.01), although the glycemic target (4.0-6.0 mmol/L) was not achieved. No hypoglycemic events occurred. Endogenous glucose production was higher during LG than SG (LG 2.9 ± 0.8 vs. SG 1.5 ± 1.1 mg/kg per minute; *P *= 0.02), due to increased glycogenolysis (LG 1.0 ± 0.6 vs. SG 0.0 ± 1.0 mg/kg per minute; *P *< 0.05). Leucine balance, indicating protein balance, was negative but not affected by glucose intake (LG -54.8 ± 14.6 vs. SG -58.8 ± 16.7 μmol/kg per hour; *P *= 0.57).

**Conclusions:**

Currently recommended glucose intakes aggravated hyperglycemia in children early after cardiac surgery with CPB. Reduced glucose intake decreased blood glucose concentrations without causing hypoglycemia or affecting protein catabolism, but increased glycogenolysis.

**Trial registration:**

Dutch trial register NTR2079.

## Introduction

Critically ill patients often develop hyperglycemia due to an acute stress response after (surgical) trauma and severe illness [1,2]. Undergoing cardiac surgery with cardiopulmonary bypass (CPB) increases the risk of developing hyperglycemia [3,4] because of the associated hyperoxia and hypothermia and increased inflammatory response induced by contact of blood with foreign material in the CPB system [5-7]. In addition, intra-operative glucose infusion contributes to hyperglycemia in children undergoing cardiac surgery [8].

Hyperglycemia in critically ill children is reported to be associated with increased morbidity and mortality [9-11]. This has led to the widespread use of insulin therapy to achieve blood glucose targets in the pediatric intensive care unit (PICU) [12]. A randomized trial in critically ill children, three quarters of whom were cardiac surgery patients, showed that at the research location intensive insulin therapy was associated with a decrease in mortality of 6% to 3% and a decreased morbidity [13]. A major drawback of this therapy was the high incidence of hypoglycemia (25%, blood glucose concentrations equal to or less than 2.2 mmol/L) [13]. Hypoglycemia has been associated with adverse outcome in the PICU [10] and may adversely affect the developing brain of young children [14-16].

An alternative approach to prevent hyperglycemia and avoid the use of insulin might be reducing intravenous (IV) glucose infusion to below current recommendations for glucose intake (approximately 5.0 mg/kg per minute) [12,17,18]. However, a reduced energy intake could result in increased protein catabolism and, subsequently, adverse outcome [19]. We hypothesized that currently recommended glucose intake in children after cardiac surgery contributes to the development of hyperglycemia and that reducing glucose intake to below these standard intakes would result in blood glucose levels in the glycemic target range of 4.0 to 6.0 mmol/L without causing hypoglycemia.

The first aim of this study was to investigate whether reducing IV glucose intake would prevent hyperglycemia in children after cardiac surgery without causing hypoglycemia. This was determined by using a randomized blinded controlled crossover design providing for both low IV glucose intake (LG 2.5 mg/kg per minute) and standard IV glucose intake (SG 5.0 mg/kg per minute). The second aim was to determine the effects of reduced glucose intake on glucose kinetics and on both leucine kinetics and albumin synthesis as indicators of protein metabolism by using stable isotope tracer methodology.

## Materials and methods

### Patients and setting

Children admitted to the Intensive Care of Erasmus MC - Sophia Children's Hospital after cardiac surgery for congenital heart disease between June 2010 and October 2010 were consecutively enrolled. Inclusion criteria were age of greater than 30 days, body weight (BW) of less than 30 kg, CPB during surgery, arterial and central venous lines, and hemodynamic stability (with or without inotropic support). Exclusion criteria were chromosomal disorder, pre-existent metabolic or endocrine disorder, liver failure, and insulin therapy at the start of the study. The medical ethical review board of Erasmus MC, Rotterdam, The Netherlands, approved this study. Prior to inclusion in the study, we obtained written informed consent from parents or legal representatives of patients.

#### Cardiac surgery

Anesthetic and peri-operative procedures have been described in detail previously [20]. Maximal arterial oxygen tension was targeted at 20 kPa. On CPB, either mild hypothermia of 28 to 32°C or circulatory arrest with deep hypothermia of 18°C nasopharyngeal temperature and 21°C rectal temperature (deep hypothermic circulatory arrest) was achieved. Antegrade cerebral perfusion was established when appropriate. Patients received 30 mg/kg methylprednisolone during surgery as standard care. Priming fluid of the CPB system contained 0.5 g/kg human albumin, and during CPB, patients received supplementary albumin to maintain a colloid oncotic pressure of greater than 15 mm Hg. Intra-operatively administered fluids did not contain glucose.

Post-operatively, IV glucose intake was provided at 4.0 to 6.0 mg/kg per minute, and total fluid intake, including medications, was restricted in the first 24 hours after surgery to 50 mL/kg per day if BW was less than 10 kg and to 750 mL/m^2 ^per day if BW was 10 to 30 kg. Patients were weaned off the ventilator when possible as standard practice. No corticosteroids were provided in the post-operative course.

### Study design and interventions

**Figure 1:**
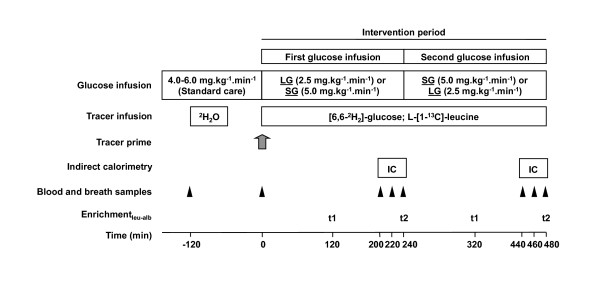
**Schematic presentation of the study in children receiving low or standard glucose intake after cardiac surgery**. In a randomized blinded crossover design, subjects received low glucose or standard glucose intake while a primed continuous stable isotope tracer protocol was conducted. The gray arrow indicates prime of tracers before continuous infusion. Black triangles indicate time points of arterial blood and breath sampling for laboratory parameters and isotopic enrichment measurements of glucose and leucine tracers. Enrichment_leu-alb _indicates the enrichment of [1-^13^C]-leucine incorporated into albumin, and 't1' and 't2' represent time points of blood sampling for determination of Enrichment_leu-alb _and calculation of fractional albumin synthesis. IC, indirect calorimetry; LG, low glucose intake; SG, standard glucose intake.

Eight hours after cardiac surgery, we started the experimental protocol, which lasted for 10 hours. See Figure 1 for the study design. Low glucose intake (LG) (2.5 mg/kg per minute) and standard glucose intake (SG) (5.0 mg/kg per minute) were provided intravenously (IV) in a crossover manner to diminish the effect of timing after cardiac surgery on metabolic variables. Randomization for the order of glucose intake was performed by means of computer-generated sealed envelopes. Indistinguishable syringes with equal volume but different glucose concentrations were prepared in order to keep fluid intake equal throughout the protocol. Laboratory personnel, nursing staff, and investigators were blinded until analyses were finished.

In the post-surgical period prior to the start of the study, glucose intake was infused as per standard care (4.0 to 6.0 mg/kg per minute). After baseline blood and breath samples were obtained, the study glucose intake (LG or SG first) was started at t = 0. Simultaneously, a primed continuous 8-hour IV stable isotope tracer infusion (described under 'Materials and sample processing') was administered. Four hours after the start of the tracer infusion (t = 240), the glucose intake was switched to the alternate level. A washout period was not deemed necessary, since glucose turnover is rapid and steady state can be achieved at 80 minutes after the start of a glucose infusion [21]; thus, carry-over effects were not expected.

Blood glucose concentrations were determined at t = 0 and at the end of both interventions (t = 240 and t = 480) along with C-reactive protein, pre-albumin, albumin, free fatty acids, triglycerides, insulin, and cortisol to describe inflammatory, metabolic, and hormonal characteristics. Blood glucose concentrations of less than 4.0 mmol/L were considered hypoglycemic; concentrations of greater than 6.0 mmol/L were considered hyperglycemic. Plasma albumin concentrations of less than 35 g/L were considered hypoalbuminemic.

Carbon dioxide production (VCO_2_), oxygen consumption (VO_2_), and respiratory quotient were determined by indirect calorimetry (Deltatrac™ II MBM-200; Datex-Ohmeda Division Instrumentarium Corp., Helsinki, Finland) in the last 40 minutes of each glucose infusion period, either by canopy mode or on the ventilator.

Severity of illness was assessed by the Pediatric Index of Mortality (PIM) score [22], the Pediatric Risk of Mortality score [23], and the pediatric logistic organ dysfunction (PELOD) score [24]. For all three, higher scores indicate higher severity of disease. Risk Adjustment for Congenital Heart Surgery [25] and Aristotle comprehensive complexity score [26] were assessed. For both, higher scores indicate increased complexity of cardiac surgery. Furthermore, vasopressor score at the start of the interventions was calculated as described by Zuppa and colleagues [27]. Estimated energy expenditure was calculated with the Schofield equation [28].

### Outcome measures

The primary outcome measure was blood glucose concentration during the interventions. Secondary outcome measures were glucose rate of appearance, endogenous glucose production (EGP), and rates of gluconeogenesis and glycogenolysis; leucine flux, leucine release from protein, leucine oxidation, non-oxidative leucine disposal, and leucine balance; whole body protein breakdown, whole body protein synthesis, and whole body protein balance; and albumin synthesis rates and contribution of albumin synthesis to whole body protein synthesis.

### Materials and sample processing

Stable isotope tracers (at least 98% enriched) were purchased from Cambridge Isotope Laboratories, Inc. (Andover, MA, USA). The hospital pharmacy of Erasmus MC, Rotterdam, The Netherlands, compounded the tracer solutions and tested them for sterility and pyrogenicity. At t = -120, ^2^H_2_O (4 g/kg) was infused intravenously over the course of 1 hour to prime the body water pool. At t = 0, a bolus of NaH^13^CO_3 _(2.1 μmol/kg) was infused to prime the bicarbonate pool followed by primed continuous administration of [6,6-^2^H_2_]-glucose (40 μmol/kg; 48 μmol/kg per hour) and L-[1-^13^C]-leucine (8 μmol/kg; 8 μmol/kg per hour) to study glucose and leucine metabolism, respectively (Figure 1).

Blood samples were obtained at standard frequent intervals (Figure 1) from the arterial line and were centrifuged (2 minutes, 2,000*g*), and plasma was frozen at -80°C until samples were analyzed. Three breath samples of approximately 15 mL of expiratory air per time point were taken from the outlet of the ventilator if patients were ventilated [29] or by the direct nasopharyngeal sampling method collecting air from a gastric tube inserted 1 to 1.5 cm in the nasopharynx [30]. The collected air was transferred to impermeable vacuum glass tubes and stored at room temperature until analysis.

### Measurements

Blood glucose concentrations and plasma albumin concentrations were determined (the former by the hexokinase method) on a Roche Modular Analytics P 800-Module (Roche Diagnostics Nederland, Almere, The Netherlands). Insulin was analyzed in blood with standard human insulin-specific radioimmunoassay techniques. C-reactive protein, pre-albumin, free fatty acids, triglycerides, and cortisol were determined by standard in-house protocols.

Enrichment of deuterated water in plasma was determined by isotope ratio mass spectrometry (Delta+XP; Thermo Fisher Scientific, Bremen, Germany). Glucose M+1 enrichment with ^2^H derived from ^2^H_2_O was analyzed by means of gas chromatography mass spectrometry (GC 6890, MS 8973; Agilent Technologies, Wilmington, DE, USA) by using the penta-acetate derivative in negative chemical ionization mode as previously described [31,32]. Glucose M+2 enrichment derived from [6,6-^2^H_2_]-glucose was determined as its aldonitrile penta-acetate derivative in electron impact ionization mode by using a slightly modified method as previously described [33]. Standard curves were prepared by mixing aqueous solutions of natural and labeled glucose for both enrichment and concentration determination. The mass spectrometric analyses were performed on a mass spectrometer coupled with a gas chromatograph (GC 7890 A, MS 5975 C; Agilent Technologies Netherlands BV, Amstelveen, The Netherlands). A chemically bonded DB-5 ms (J&W Scientific, Folsom, CA, USA) capillary column with a length of 30 m, an internal diameter of 0.25 mm, and a film thickness of 0.25 μm was used for the chromatographic separation. The intensities of the 187.2 and 189.2 fragments were selected for measurement of, respectively the non-enriched and the 6,6-^2^H_2_-enriched aldonitrile penta-acetate derivative of glucose. All measurements were carried out in selective ion monitoring mode. Leucine kinetics was calculated from plasma alpha-ketoisocaproate (α-KIC) M+1 enrichment that was determined by gas chromatography mass spectrometry after derivatization to butyldimethyl-silylquinoxalinol derivatives [34]. Breath samples were analyzed for enrichment of ^13^CO_2 _by using an infrared isotope analysis technique (Helifan; Fischer Instruments, Leipzig, Germany). ^13^C enrichment was expressed as atom percentage excess above baseline for subsequent calculation of leucine oxidation [35]. The enrichment of incorporated leucine in albumin was determined on a gas chromatograph-combustion-isotope ratio mass spectrometer (Delta XP; Thermo Fisher Scientific) as described before [36]. Plasma samples were analyzed as triplicates; breath samples were collected in triplicate and analyzed once.

### Calculations

Glucose kinetics was estimated by using the Steele equation [37], based upon the final 40 minutes of both glucose infusion periods (steady state); whole body leucine kinetics was calculated by conventional isotope dilution equations by using a stochastic model [38]. At steady-state plateau, rate of appearance (Ra) equals the rate of disappearance (Rd) as follows:

(1) $$\text{Ra = Rd }=i\times \left[\left({\text{E}}_{\text{inf}}/){\text{E}}_{\text{pl}}\right)\;-\text{ 1}\right],$$

where *i *is the infusion rate of the labeled tracer, E_inf _is the tracer enrichment of the infusate, and E_pl _is the tracer enrichment in plasma.

#### Glucose kinetics

Plasma [6,6-^2^H_2_]glucose enrichment (in mole percent excess) and the exogenous glucose infusion rate were used for data calculation. Under steady-state conditions, total glucose rate of appearance is equal to the rate of disappearance [37], the latter of which reflects glucose utilization. Rates of EGP, glucose clearance, glycogenolysis, and gluconeogenesis were calculated as previously described [21,39,40].

EGP rate was calculated as follows:

(2) $$\text{EGP }=\text{ R}{\text{a}}_{\text{Glucose}}-\text{ GIR},$$

where GIR is the total glucose infusion rate in mg/kg per minute.

Fractional gluconeogenesis was calculated as previously described [32]. Briefly, the average enrichment of ^2^H on each glucose carbon was calculated with the following equation:

(3) $$\text{Average }\left(\text{M}+\text{1}\right)\text{d }=\;\left(\text{M}+\text{1}\right){\text{d}}_{\left(m/)z\;\text{169}\right)}/)\text{6},$$

where (M+1)d_(*m/z *169) _is the M+1 enrichment of deuterium of glucose measured by using *m/z *170/169 and '6' is the number of ^2^H labeling sites on the *m/z *169 fragment of glucose.

Because body water is the precursor pool for deuterium or hydrogen, the extent of deuterium labeling of glucose during the gluconeogenic process when ^2^H_2_O is infused is a measure of fractional gluconeogenesis. Therefore, with the average deuterium enrichment in *m/z *170/169 for calculating fractional gluconeogenesis (FracGNG), the equation is

(4) $$\text{FracGNG = average }\left(\text{M}+\text{1}\right)\text{d}/{\text{E}}_{\text{H2O}},$$

where E_H2O _is the deuterium enrichment in body water.

The absolute rate of appearance of plasma glucose from gluconeogenesis (Ra_GNG_) and glycogenolysis were calculated:

(5) $$\text{Gluconeogenesis }=\text{ R}{\text{a}}_{\text{Glucose}}\times \text{FracGNG}$$

(6) Glycogenolysis = EGP - Gluconeogenesis

Glucose clearance, as a measure of the disposal of glucose per unit of blood glucose, was calculated with the following equation [21,40]:

(7) $$\text{Glucose clearance}=\text{R}{\text{a}}_{\text{glucose}}/)\left({\text{C}}_{\text{glucose}}\times 0.\text{18}\right),$$

where glucose clearance is expressed in mL/kg per minute, C_glucose _is the glucose concentration in blood in mmol/L, and 0.18 the factor to convert the concentration to mg/mL.

#### Leucine kinetics

Plasma leucine kinetics, which is indicative of whole body protein kinetics, was calculated as follows. Whole body leucine fluxes (Ra_Leu_) (μmol/kg per hour) were calculated according to Equation 1 from [^13^C]α-ketoisocaproate ([^13^C]α-KIC) as previously described [34,41,42].

Leucine release from protein (LRP), which is indicative of protein breakdown, was calculated as follows:

(8) $$\text{LRP }=\text{ R}{\text{a}}_{\text{Leu}}-i,$$

where *i *represents the tracer infusion rate.

Leucine oxidation rates (μmol/kg per hour) were calculated with the following equation:

(9) $$\text{LeucineOx }=\text{ VC}{\text{O}}_{\text{2}}\times \left({\text{E}}^{\text{13}}\text{C}{\text{O}}_{\text{2}}/)\text{69}.\text{18}\right)/[{}^{\text{13}}\text{C}]\alpha -\text{KIC},$$

where 69.18 is the ^13^CO_2 _refraction correction factor for critically ill children [35]. VCO_2 _is measured in mL/minute and converted to mmol/hour^2 ^by multiplying by 60 minutes and dividing by 22.4 L/mol. The latter is the volume of one mole of an ideal gas at standard temperature and pressure.

Non-oxidative leucine disposal (NOLD) (leucine used for protein synthesis, which is indicative of protein synthesis) was calculated as follows:

(10) $$\text{NOLD }=\text{ R}{\text{a}}_{\text{leu}}-\text{ LeucineOx}$$

Leucine balance (μmol/kg per hour) was calculated as follows:

(11) Leucine balance = LRP - NOLD.

#### Protein kinetics

Whole body protein turnover was calculated from the model described by Golden and Waterlow [43]. To convert leucine kinetics into protein kinetics, we assumed that the average content of leucine in human proteins was 621 μmol/g [44]. Thus, leucine kinetics in μmol/kg per hour was divided by 621 μmol/g and multiplied by 24 hours to derive protein kinetics in g/kg per day (protein synthesis from NOLD, protein breakdown from LRP, and protein balance from leucine balance).

#### Albumin synthesis

By measuring the incorporation of [1-^13^C]-leucine in albumin, we calculated the fractional and absolute synthesis rates of albumin and the contribution of albumin synthesis to the whole body protein synthesis. Fractional albumin synthesis rate (FSR) represents the renewed fraction of the intravascular albumin pool per time unit (percentage per day) and was calculated as follows [45]:

(12) $$\text{FSR}=\left({\text{E}}_{\text{leu}-\text{alb},\text{ t}2}-{\text{E}}_{\text{leu}-\text{alb},\text{ t}1}\right)/){\text{E}}_{\alpha -\text{KIC}}\times \left(24\times 60\right)/)\left(\text{t}2-\text{t}1\right)\times 100\%,$$

where E_leu-alb _is the enrichment (mole percent excess) of incorporated leucine in albumin at t1 (t = 120 and t = 360 for the first and second glucose infusions, respectively) and t2 (t = 240 and t = 480 for the first and second glucose infusions, respectively) (Figure 1). E_α-KIC _is the mean enrichment of the precursor, i.e. plasma α-KIC, at these time points in mole percent excess.

The absolute albumin synthesis rate (ASR) (mg/kg per day) was calculated as follows [45]:

(13) $$\text{ASR }=\text{ FSR}\times {\text{C}}_{\text{alb}}\times \text{vo}{\text{l}}_{\text{bl}}\times \left(\text{1}-\text{Ht}\right)\times \text{weigh}{\text{t}}^{-\text{1}},$$

where C_alb _is plasma albumin concentration (g/L), vol_bl _is the total volume of blood in the body (for these subjects assumed to be 75 mL/kg), Ht is hematocrit, and (1-Ht) is the fraction of blood that is plasma.

Furthermore, we calculated the contribution (percentage) of albumin ASR to whole body protein synthesis by determining the ratio of leucine incorporated into albumin to the total amount of leucine used for protein synthesis [45]:

(14) $$\text{Contribution }=\left[\left(\text{ASR}\times 0.\text{1}0\text{4}\right)/)\left(\text{NOLD}\times \text{131}.\text{2}\times \text{24}\times 0.00\text{1}\right)\right]\times \text{1}00\%,$$

where 0.104 is the mass fraction of leucine residues in albumin, 131.2 is the molecular mass of leucine, 24 is the factor to convert to days, and 0.0001 is the factor to convert to milligrams.

### Data analysis

Power analysis showed that inclusion of eight subjects with complete data would suffice to detect a statistically significant difference of 20% in plasma glucose concentrations (80% power, type I error of 5%) on the basis of baseline blood glucose levels of 7.3 mmol/L and target levels of less than 6.0 mmol/L. The Shapiro-Wilk normality test was used to determine whether data were normally distributed. Data are presented as mean ± standard deviation; non-parametric data are presented as median (interquartile range). Data during the two different glucose infusions were compared by either the paired samples *t *test (normal distribution) or the Wilcoxon matched pairs test with exact significance (non-normal distribution). Differences between subsets of subjects were assessed by the independent samples *t *test (normal distribution) or Mann-Whitney *U *test (non-normal distribution). Correlations between baseline characteristics and the primary outcome measure were determined with Spearman's rho correlation coefficient. Statistical significance was defined as a *P *value of less than 0.05. Statistical analyses were carried out with IBM SPSS Statistics version 17.0 (IBM Corporation, Armonk, NY, USA).

## Results

### Patients

We conducted the study protocol in 11 children (8 males and 3 females). In 11 subjects, blood glucose concentrations were available during both glucose infusion periods. Owing to technical problems, glucose kinetics data were collected in 9 of 11 patients. Leucine kinetics data were available in 8 of 11 patients because of the inability to conduct indirect calorimetry in all patients. Median BW was 6.8 kg (7.1 kg). Mean PIM score was 12.6% ± 7.2% predicted mortality, median PRISM score was 7.5% (25.6%) predicted mortality, and median PELOD score was 1.3% (1.2%) predicted mortality. Table 1 lists other baseline characteristics.

**Table 1:** Patient characteristics of 11 children after cardiac surgery

Patient	First glucose infusion (LG or SG)	Age, months	Diagnosis and surgical intervention	RACHS-1 category	Comprehensive Aristotle complexity **score**^**a**^	CPB time, hours:minutes	Aorta clamp time, hours:minutes	Vasopressor **score**^**b**^	Extubation before start of intervention period
1	LG	60.0	Sinus venosus defect patch repair	1	3.0	1:11	0:53	0	Yes
2	LG	23.3	PCPC for univentricular heart	2	6.8	0:40	0:00	0	Yes
3	LG	4.7	VSD repair	2	7.0	1:33	1:08	0	Yes
4	LG	20.6	Redo RVOT procedure after correction of TOF	2	8.5	1:55	0:59	0	Yes
5	LG	4.8	CAVSD repair	3	9.0	2:18	1:47	0	Yes
6	LG	2.5	Biventricular repair of HLHS with DHCA after hybrid preparation^c^	6	17.0	3:44	1:58	0	No
7	SG	11.7	ASD-II repair	1	3.0	0:37	0:16	0	Yes
8	SG	24.4	Sinus venosus defect patch repair	1	3.0	1:23	0:57	0	Yes
9	SG	3.1	VSD repair	2	7.0	1:22	0:47	0	No
10	SG	2.6	TOF repair with transannular patch	2	8.0	1:14	0:52	0	Yes
11	SG	5.2	Biventricular repair of HLHS with DHCA after hybrid preparation^c^	6	17.0	4:32	2:33	7	No
LG as first glucose infusion, median (IQR) or mean ± SD	**-**	12.7 (28.3)	-	2 (3)	7.3 (5.3)	1:44 (1:36)	1:07 ± 0:42	0 (0)	-
SG as first glucose infusion, median (IQR) or mean ± SD	**-**	5.1 (15.3)	-	2 (2)	6.0 (9.5)	1:22 (2:02)	1:05 ± 0:51	0 (3.5)	-
All, median (IQR) or mean ± SD	**-**	5.1 (20.2)	-	2 (2)	6.0 (4.0)	1:23 (1:07)	1:06 ± 0:44	0 (0)	-

Normally distributed data (as assessed by Shapiro-Wilk normality test) are presented as mean ± standard deviation (SD), and non-normally distributed data are presented as median (interquartile range, or IQR). There were no significant differences between patients receiving low glucose intake first or standard glucose intake first (Mann-Whitney *U *test). ^a^Comprehensive Aristotle complexity score [26]; ^b^vasopressor score [27]; ^c^deep hypothermic circulatory arrest (DHCA) times (hours:minutes) were 0:53 and 1:31 for patients 6 and 11, respectively; antegrade cerebral perfusion times as part of DHCA were 0:32 and 1:21 for patients 6 and 11 respectively. ASD-II, ostium secundum atrium septal defect; CAVSD, complete atrial ventricular septal defect; CPB, cardiopulmonary bypass; HLHS, hypoplastic left heart syndrome; LG, low glucose intake (2.5 mg/kg per minute); PCPC, partial cavo-pulmonar connection; RACHS-1, risk adjusted congenital heart surgery score [25]; RVOT, right ventricle outflow tract; SG, standard glucose intake (5.0 mg/kg per minute); TOF, tetralogy of Fallot; VSD, ventricular septal defect.

There were no clinically important or statistical differences in baseline characteristics between patients randomly assigned to start with LG and those who started with SG (Table 1). All patients received prophylactic antibiotics (cefazolin), diuretics, morphine, and/or acetaminophen for pain relief. One patient was ventilated with nitric oxide for pulmonary hypertension but was hemodynamically stable without inotropes. Other drugs administered included norepinephrine (*n *= 1), milrinone (*n *= 2), and IV nitroglycerine (*n *= 2). See Table 1 for vasopressor scores at the start of the study protocol. The first glucose infusion was started a mean of 9.5 ± 1.9 hours after cardiac surgery (t = 0). During LG, glucose intake including glucose tracers was 2.6 ± 0.3 mg/kg per minute; during SG, glucose intake including glucose tracers was 5.0 ± 0.4 mg/kg per minute (*P *< 0.001; paired samples *t *test).

### Blood glucose concentrations and laboratory parameters

Blood glucose concentrations were significantly lower during LG than during SG (Table 2). On average, the glycemic target (4.0 to 6.0 mmol/L) was not achieved during either of the glucose infusions. No hypoglycemic events occurred, and the lowest blood glucose concentration measured was 6.2 mmol/L. Table 2 lists other metabolic characteristics.

**Table 2:** Metabolic characteristics of children receiving low or standard glucose intake after cardiac surgery

Metabolic characteristics	Before experiment	Low glucose intake (2.5 mg/kg per minute)	Standard glucose intake (5.0 mg/kg per minute)	*P *value
Glucose intake	3.6 ± 0.7	2.6 ± 0.3	5.0 ± 0.4	< 0.001
Blood glucose, mmol/L	9.5 ± 2.0	7.3 ± 0.7	9.3 ± 1.8	0.007
Estimated energy expenditure, kcal/kg per day^a^	54.7 ± 5.8			
Energy intake, kcal/kg per day		12.1 ± 1.3	23.5 ± 2.1	< 0.001
Measured energy expenditure, kcal/kg per day		44.9 ± 10.9	46.1 ± 10.7	0.856
VCO_2_, mL/kg per minute^b^		5.6 ± 1.3	5.7 ± 1.2	0.901
VO_2_, mL/kg per minute^b^		6.4 ± 1.7	6.6 ± 1.5	0.813
Respiratory quotient		0.87 (0.21)	0.89 (0.06)	0.719
C-reactive protein, mg/L	13 ± 7	32 ± 17	32 ± 16	0.933
Pre-albumin, g/L	0.18 (0.04)	0.18 (0.03)	0.17 (0.03)	0.203
Albumin, g/L	38 ± 5	38 ± 4	38 ± 5	1.000
Triglycerides, mmol/L	0.41 (0.32)	0.41 (0.41)	0.47 (0.35)	0.687
Free fatty acids, mmol/L	0.71 ± 0.23	0.66 ± 0.13	0.53 ± 0.12	0.013
Cortisol, nmol/L	535 ± 193	229 ± 100	208 ± 42	0.429
Insulin, pmol/L	90 (229)	61 (83)	142 (199)	0.064
Insulin/glucose ratio, pmol/mmol	0.6 (1.1)	9.0 (13.5)	17.8 (20.8)	0.105

*P *values indicate statistical comparison between glucose intakes (low glucose intake and standard glucose intake) only. Normally distributed data (as assessed by Shapiro-Wilk normality test) are presented as mean ± standard deviation, and comparison between glucose intakes was done by paired samples *t *test. Non-normally distributed data are presented as median (interquartile range), and comparison between glucose intakes was done by Wilcoxon matched pairs test. ^a^Estimated with Schofield equation [28]; ^b^*n *= 8 for carbon dioxide production (VCO_2_), oxygen consumption (VO_2_), and respiratory quotient; for other variables, *n *= 11.

### Glucose kinetics

**Figure 2:**
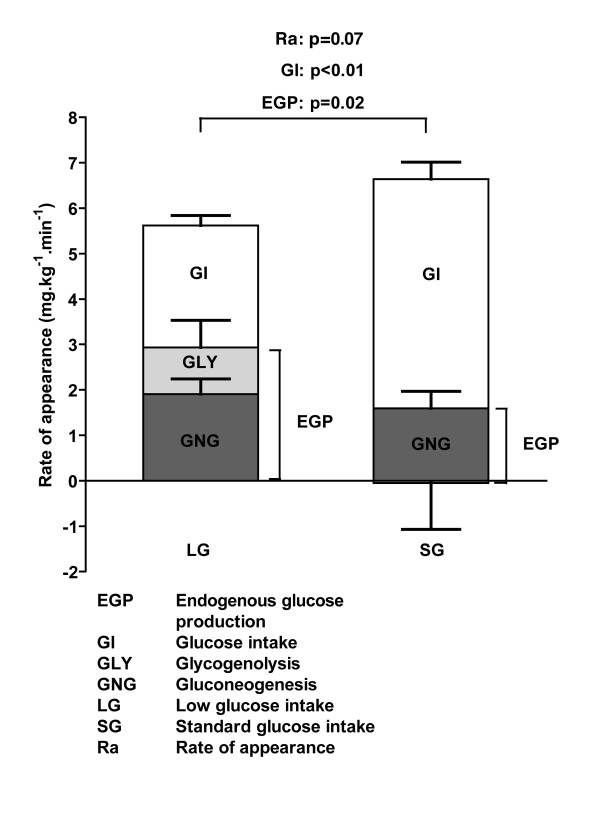
**Glucose kinetics in children receiving low or standard glucose intake after cardiac surgery**. Data are presented as mean ± standard deviation in mg/kg per minute in stacked bars (*n *= 9). Error bars are shown for components of rate of appearance of glucose only: glucose intake (GI), glycogenolysis (GLY), and gluconeogenesis (GNG). Comparison between glucose intakes was done by paired samples *t *test. Entire stacked bars represent rate of appearance of glucose, which consists of exogenous glucose intake and endogenous glucose production. The latter is composed of gluconeogenesis and glycogenolysis. Glycogenolysis during standard glucose intake was not significantly different from zero (*P *= 0.89; one sample *t *test). EGP, endogenous glucose production; LG, low glucose intake; Ra, rate of appearance; SG, standard glucose intake.

Steady-state ^2^H_2_O enrichments were 0.72 ± 0.06 and 0.72 ± 0.07 atom percent excess during the first and second glucose infusions, respectively. During SG, EGP was not fully suppressed and consisted entirely of gluconeogenesis, while glycogenolysis did not differ from zero (*P *= 0.89; one sample *t *test) (Table 3 and Figure 2). During LG, glucose rate of appearance tended to be lower, with a significantly higher EGP than during SG. The higher EGP during LG resulted from increased glycogenolysis, while gluconeogenesis was maintained at the same rate as during SG (Table 3 and Figure 2).

**Table 3:** Glucose, leucine, and albumin kinetics in children receiving low or standard glucose intake after cardiac surgery

	Low glucose intake (2.5 mg/kg per minute)	Standard glucose intake (5.0 mg/kg per minute)	*P *value
Glucose kinetics (*n *= 9)^a^			
Glucose Ra, mg/kg per minute	5.6 ± 0.9	6.6 ± 1.1	0.071
Fractional gluconeogenesis as percentage of Ra	34 ± 3	24 ± 5	0.002
Glucose clearance rate, mL/kg per minute	4.19 ± 0.54	4.03 ± 0.64	0.362
Leucine kinetics, μmol/kg per hour (*n *= 8)			
Leucine Ra	195.2 ± 21.2	209.3 ± 27.3	0.218
Leucine oxidation	63.1 ± 14.6	68.0 ± 15.4	0.573
Leucine release from protein^a^	187.0 ± 20.9	201.1 ± 27.3	0.218
Non-oxidative leucine disposal^b^	132.1 ± 17.7	141.3 ± 35.5	0.496
Leucine balance	-54.8 ± 14.6	-59.8 ± 15.8	0.573
Albumin synthesis (*n *= 8)			
Fractional albumin synthesis rate, percentage per day	9.2 ± 3.5	9.6 ± 4.0	0.756
Absolute albumin synthesis rate, mg/kg per day	157.3 (94.6)	139.5 (111.3)	0.742
Contribution to total protein synthesis, percentage	4.2 ± 1.3	4.2 ± 1.6	0.976

Normally distributed data (as assessed by Shapiro-Wilk normality test) are presented as mean ± standard deviation, and comparison between glucose intakes - low glucose intake and standard glucose intake - was done by paired samples *t *test. Non-normally distributed data are presented as median (interquartile range), and comparison between glucose intakes was done by Wilcoxon matched pairs test. ^a^Indicative of protein breakdown; ^b^indicative of protein synthesis. Ra, rate of appearance.

### Leucine kinetics and whole body protein metabolism

VCO_2_, VO_2_, and respiratory quotient did not differ significantly between the two glucose infusions (Table 2). Respiratory quotient values were within the normal range (0.85 to 1.00). Leucine and protein kinetics did not differ significantly between the two glucose infusions (Table 3). Whole body protein kinetics as derived from leucine kinetics were as follows for LG and SG, respectively: whole body protein breakdown 7.6 ± 0.8 versus 8.2 ± 1.1 g/kg per day (*P *= 0.22, paired samples *t *test) and whole body protein synthesis 5.4 ± 0.7 versus 5.7 ± 1.4 g/kg per day (*P *= 0.46, paired *samples t *test). Whole body protein balance was negative during both interventions but was not further aggravated by reduced glucose infusion (LG: -2.2 ± 0.6; SG: -2.4 ± 0.6 g/kg per day; *P *= 0.57; paired samples *t *test).

Patients had normal plasma albumin concentrations (Table 2). Fractional and absolute albumin synthesis rates did not differ between the two glucose infusions (Table 3). Protein synthesis consisted for 4% of albumin synthesis during both interventions.

### Correlations and subanalysis

Age, weight, height, severity of illness scores and complexity of cardiac surgery scores, CPB time, and aorta clamp time or time after surgery of starting the first glucose infusion were not correlated with blood glucose concentrations during LG and SG. Two subjects underwent deep hypothermic circulatory arrest, which is different from other cardiac surgical interventions on CPB. Subanalysis without these two patients revealed blood glucose concentrations of LG 7.4 ± 0.7 versus SG 9.3 ± 1.5 (*P *< 0.01) with the paired samples *t *test. Glucose and leucine kinetics were not affected, apart from slightly changing the significance level of glycogenolysis (LG 1.0 ± 0.6 versus SG 0.1 ± 1.0; *P *= 0.06; paired samples *t *test).

## Discussion

Our study showed that currently recommended glucose intakes aggravated hyperglycemia in children admitted to the PICU in the first 24 hours after cardiac surgery with CPB. Furthermore, reduced glucose intake resulted in decreased blood glucose concentrations and not in hypoglycemia or increased protein catabolism. However, in contrast with our hypothesis and our previous study in healthy children undergoing elective craniofacial surgery, the glycemic target (4.0 to 6.0 mmol/L) was not achieved with reduced glucose intake [39]. In addition, it resulted in increased EGP due to increased glycogenolysis.

In recent years, the focus on intensive insulin therapy in critically ill children has increased, especially after Vlasselaers and colleagues [13] showed that in their setting this therapy resulted in decreased morbidity and mortality. However, hypoglycemia of equal to or less than 2.2 mmol/L was observed in a quarter of patients. It is considered a serious complication potentially leading to neurological damage in the long term [16]. We and others therefore suggest that insulin therapy be started at a higher glycemic threshold of approximately 8 mmol/L [12,46]. In the present study, currently recommended glucose intakes (iatrogenically) aggravated hyperglycemia, making patients eligible for insulin therapy on the basis of this threshold. We therefore postulate reduced glucose intake as the initial step to prevent hyperglycemia in the early post-operative phase. We acknowledge that this alternative approach bypasses the non-metabolic (for example anti-inflammatory and anti-apoptotic) beneficial effects of insulin [12,47]. However, within 24 hours after cardiac surgery, most children show spontaneous resolution of hyperglycemia [48]. The duration of insulin therapy in this population is therefore often brief. It is questionable whether beneficial effects can then be exerted and whether they outweigh the risk of hypoglycemia. In addition, insulin therapy seems to reduce morbidity and mortality predominantly by preventing hyperglycemia rather than by a direct effect of insulin [49]. Also, hyperglycemia causes cell damage, which is normally cleared by the process of autophagy, but the latter is suppressed by nutrient intake [49]. It has therefore been suggested that, when tight glycemic control is not feasible in clinical practice, moderate hyperglycemia might be tolerated when nutrient intake is restricted [49]. Therefore, reduced glucose intake seems even more promising to bridge the brief hyperglycemic period after pediatric cardiac surgery. However, since the study population was heterogeneous and small, our study provides mostly a mechanistic view of this approach. Also, our patients were relatively stable and intra-operative management in our center includes high-dose opioids to suppress the acute stress response [20]. Therefore, caution should be taken when generalizing our data to longer periods of reduced glucose intake and to different and more critically ill populations. Thus, clinical outcome studies are warranted to formulate suitable recommendations of glucose intake.

Our study is one of few studies providing data on glucose kinetics and glucose intake in critically ill children. Glucose intake of 2.5 mg/kg per minute resulted in increased EGP through increased glycogenolysis, despite hyperglycemia. The latter suggests that LG did not meet the metabolic needs of the liver. During SG, EGP was sustained, whereas in healthy individuals increased glucose intake reduces EGP [21,50-53]. These features can be explained by the metabolic stress response, which is characterized by increased EGP due to increased counter-regulatory hormones [47], impairment of insulin-induced suppression of EGP (central insulin resistance), and impairment of insulin-mediated glucose uptake (peripheral insulin resistance) [1,2,47]. Since cortisol concentrations were normal, inotropic support was limited to one patient, and all patients received corticosteroids, unsuppressed EGP (gluconeogenesis) most likely resulted from insulin resistance. Craniofacial surgery patients in whom we observed unsuppressed EGP as well did achieve the glycemic target when receiving LG [39]. Their insulin resistance was possibly less pronounced, as supported by lower insulin concentrations, lower insulin/glucose ratios, and higher glucose clearance rates (*n *= 8, 5.0 ± 1.4 mL/kg per minute; unpublished data) [39]. In the present study, insulin resistance seemed to be higher during SG than LG, as shown by a higher insulin/glucose ratio, which approached the threshold of a hyperinsulinemic response (18 pmol/mmol) [54]. The lack of statistical significance might be explained by the small sample size. In adults, increased insulin resistance is associated with increased risk of post-operative complications after major surgery [55]. Therefore, glucose solutions in the first day after major surgery in adults should be avoided [55]; in young children, this would translate to avoiding SG.

We did not find adverse effects of LG on protein metabolism or albumin synthesis rates, and this is consistent with previous studies from our group [36,39,56]. Plasma albumin levels were in the normal range and in agreement with previous reports in children receiving human albumin during CPB [57,58]. In contrast, infants after craniofacial surgery and septic adolescents, in whom we studied albumin kinetics previously, were hypoalbuminemic. This might explain why they showed higher albumin synthesis rates than the current cardiac patients [36]. Albumin synthesis rates were not affected by glucose intake, protein intake, or insulin administration in any of the groups [36]. Possibly owing to its relatively short duration (240 minutes), we did not find increased protein catabolism during LG. At the time of the study, patients likely had substantial glycogen stores. With prolonged low glucose intake, glycogen stores might eventually be depleted, further triggering gluconeogenesis and protein catabolism to provide amino acids as gluconeogenic substrate. Whether further reducing glucose intake is more effective to reduce blood glucose concentrations and what the repercussions are on glucose and protein metabolism therefore need to be investigated.

There are some limitations to this study. First, glycogenolysis rates in some patients were negative during SG, but this is physiologically not possible. This may have resulted from an underestimation of EGP because of dilution of the tracer pool by re-uptake of newly produced glucose in the liver as a consequence of hepatic intralobular functional heterogeneity [59]. Second, we did not measure cerebral glucose uptake as the lower limit [17] and glucose oxidation rates as the upper limit [18] of glucose intake. Since hypoglycemia was not apparent in our population, we assume that cerebral glucose uptake was not impaired during reduced glucose intake. We refrained from measuring glucose oxidation with [^13^C]-glucose, because our [1-^13^C]-leucine tracer would have interfered with ^13^CO_2 _measurements for glucose oxidation.

## Conclusions

Glucose intake at currently recommended rates in the initial phase of post-operative care in the PICU aggravated hyperglycemia in children younger than 6 years and with a BW of less than 30 kg after cardiac surgery. Reducing glucose intake to 2.5 mg/kg per minute resulted in decreased blood glucose concentrations without causing hypoglycemia or increased protein catabolism. Reduced glucose intake might be feasible as an initial step targeting hyperglycemia in the early post-operative course of cardiac surgery in relatively stable children, potentially avoiding insulin use and its complications. We acknowledge that we cannot extrapolate our results to longer durations of glucose infusions or different patient populations. The concept of reduced glucose intake as an alternative to insulin therapy seems promising, however, and deserves further investigation in these settings.

## Key messages

• Currently recommended glucose intake (5.0 mg/kg per minute) aggravated hyperglycemia (≥ 6 mmol/L) in children in the early post-operative phase after cardiac surgery.

• Reducing glucose intake to 2.5 mg/kg per minute reduced blood glucose levels without causing hypoglycemia.

• Reducing glucose intake did not increase protein catabolism.

• The increased endogenous glucose production during reduced glucose intake resulted from increased glycogenolysis, while gluconeogenesis was maintained at the same rate, as compared with standard glucose intake.

• Reducing glucose intake might be used as an initial step to prevent hyperglycemia in the early post-operative phase after cardiac surgery in children weighing less than 30 kg in body weight.

## Abbreviations

ASR: absolute albumin synthesis rate; BW: body weight; CPB: cardiopulmonary bypass; EGP: endogenous glucose production; FSR: fractional albumin synthesis rate; IV: intravenous; LG: low glucose intake; LRP: leucine release from protein; NOLD: non-oxidative leucine disposal; PELOD: pediatric logistic organ dysfunction; PICU: pediatric intensive care unit; PIM: Pediatric Index of Mortality; PRISM: Pediatric Risk of Mortality; Ra: rate of appearance; Rd: rate of disappearance; SG: standard glucose intake; VCO_2_: carbon dioxide production; VO_2_: oxygen consumption.

## Competing interests

The authors declare that they have no competing interests.

## Authors' contributions

CTdB participated in the design of the study, carried out the study, analyzed data, and was the primary author of the manuscript. SCV participated in the design of the study, participated in carrying out the study, analyzed data, and helped to draft the manuscript. HS and SKC provided essential technical support and performed sample analysis. AJB, JBvG, and KFJ participated in the design of the study and the interpretation of data and helped to draft the manuscript. All authors read and approved the final manuscript.
